# CAFs-derived SPI1 in tumor fibroblasts promotes malignant behaviors of liver cancer cells and immune escape by regulating HRAS and PD-L1 transcription

**DOI:** 10.1186/s41065-025-00605-2

**Published:** 2025-11-26

**Authors:** Ke Miao, Jiaren Zhou, Jieyun Chen

**Affiliations:** 1 Department of Hepatobiliary Surgery, Yiwu Central Hospital, No.519 Nanmen Street, Yiwu, Zhejiang 322000 China; 2 Department of Critical Care Medicine, Xingtai Central Hospital, Xingtai City, Hebei 054000 China; 3 Department of Neurology Ward 6, Xingtai Central Hospital, Xingtai City, Hebei 054000 China

**Keywords:** Hepatocellular carcinoma, CAFs, SPI1, Immune escape, HRAS/PD-L1 signal pathway

## Abstract

**Background:**

As the fifth most common cancer worldwide, hepatocellular carcinoma (HCC) is a highly malignant disease with a formidable prognosis. Within the tumor microenvironment, cancer-associated fibroblasts (CAFs) play a paramount role in tumorigenesis and progression by providing a supportive environment for cancer cells. This research aims to elucidate the regulatory mechanisms of CAFs and salmonella pathogenicity island 1 (SPI1) in HCC progression.

**Methods:**

Protein and mRNA expression levels were determined by western blot and reverse-transcription quantitative polymerase chain reaction (RT-qPCR), respectively. The ability of glycolysis was detected using the glucose consumption and lactate production test kit. Cell proliferation was examined using colony formation assay. Cell migration and invasion were assessed via wound healing and transwell assays, respectively. The cell apoptosis was examined by flow cytometry and terminal deoxynucleotidyl transferase dUTP Nick-End Labeling (TUNEL).

**Results:**

The CAFs and para-cancer fibroblasts (PAFs) were successfully separated. CAFs under hypoxic stress (H/CAFs) promoted glucose consumption and lactate production in HCC cells (Huh7 and Hep3B). CAFs conditioned medium (CAFs-CM) facilitated HCC cell proliferation, migration, invasion, glucose consumption, and lactate production. CAFs-CM facilitated programmed cell death 1 ligand 1 (PD-L1) and CD8^+^T apoptosis and hindered proliferation of CD8^+^T. SPI1 was identified as the common target of the differentially expressed genes (DEGs) in CAFs (GSE192912) and transcription factors (TF). The mRNA and protein expression of SPI1 was increased in CAFs. SPI1 knockdown suppressed PD-L1 expression levels, glucose consumption, lactate production, proliferation, invasion, and immune escape in CAFs-CM-cultured HCC cells.

**Conclusions:**

SPI1 derived from CAFs facilitates the malignant behaviors of HCC cells by up-regulating v-Ha-ras Harvey rat sarcoma viral oncogene homolog (HRAS) expression. This study enriches our understanding of HCC at the molecular level and may pave the way for the development of a novel strategy for the treatment of HCC.

**Supplementary Information:**

The online version contains supplementary material available at 10.1186/s41065-025-00605-2.

## Introduction

Hepatocellular carcinoma (HCC) is one of the most common and lethal cancers [1]. It is characterized by aggressive and frequent late-stage diagnosis, which, coupled with limited therapeutic options, contributes to a five-year survival rate below 20% in most countries [2, 3]. Accounting for approximately 85% of primary liver cancer, HCC represents the predominant histological subtype encountered clinically [4]. Although surgical resection remains the most effective treatment, tumor progression continues to be a major determinant of poor prognosis in HCC patients [5, 6]. Consequently, identifying key drivers of HCC progression and elucidating their underlying mechanisms are essential for improving clinical outcomes.

Tumor microenvironment (TME) is a complex ecosystem comprising diverse cellular elements, including fibroblasts and immunocytes, as well as stromal components [7]. Within the TME, cancer-associated fibroblasts (CAFs) represent a predominant cell population and play a critical role in facilitating cancer metastasis and malignant progression [8, 9]. In HCC, CAFs contribute to tumor initiation and development via paracrine signaling involving multiple inflammatory cytokines and chemokines [10–12]. Specifically, CAFs have been shown to enhance migration, invasion, proliferation, remodeling of the TME, and drug resistance in HCC [13, 14]. Interleukin-6, for instance, regulates the polarization of M2-type macrophages, suggesting a potential mechanism by which CAFs-conditioned medium (CAFs-CM) induces the phenotypic shift of M0 macrophages toward a tumor‑associated macrophage phenotype [15]. Nevertheless, the precise mechanisms underlying the crosstalk between CAFs and HCC cells remain intricate and not fully elucidated.

Salmonella pathogenicity island 1 (SPI1), a member of E26 transformation-specific (ETS) transformation factor family, is critical for B cell development and broader immune cell functions [16]. In cancer biology, SPI1 contributes to tumor progression across various contexts. For instance, SPI1-mediated transcriptional activation of centrosomal protein 55 facilitated the proliferation, invasion of triple-negative breast cancer cells as well as M2 macrophage polarization [17]. The lncRNA wilms tumor 1 antisense RNA overexpression could inhibit cervical cancer progression by suppressing phosphoinositide-3-kinase adaptor protein 1 (PIK3AP1) via recruitment of SPI1 [18]. Despite these findings in other malignancies, the specific role of SPI1 in regulating HCC progression and modulating the immune microenvironment requires further elucidation.

This study aims to investigate the role of CAFs and the transcription factor SPI1 in HCC progression and immune escape. This study first demonstrates CAFs-derived SPI1 in mediating HCC cell invasion, migration, proliferation, and immune escape. Furthermore, our research may provide a viable therapeutic target for the design of innovative and more effective treatment strategies against HCC.

## Materials and methods

### Ethics approval

Written informed consent was obtained from all patients involved in this study. All protocols were approved by the Ethics Committee of Yiwu Central Hospital.

### Isolation, culture, and treatment of fibroblasts

HCC tissues and matched adjacent non-tumor tissues were collected from patients undergoing surgical resection. Para-cancer fibroblasts (PAFs) and CAFs were isolated from adjacent non-tumor tissues and HCC tissues, respectively. Briefly, small pieces (1 mm^3^) of human HCC tissues or non-tumor tissues adjacent to the HCC were digested in Dulbecco’s modified eagle’s medium (DMEM) (BersinBio, Guangzhou, China) supplemented with fetal bovine serum (10%, FBS) (Biao Leibo, Beijing, China), hyaluronidase (100 U/mL, Beyotime, Shanghai, China), collagenase type I (1 mg/mL, Gibco, Carlsbad, CA, USA), and penicillin-streptomycin (1%, Gibco) for 8 h at 37℃. Following digestion, the cell suspension was filtered through a 40 μm cell filter and centrifuged (600 rpm) for 6 min to separate fibroblasts and epithelial cells. The supernatant containing fibroblasts was centrifuged (800 rpm) for 10 min and the fibroblasts were washed twice. Next, the fibroblasts (CAFs and PAFs) were resuspended in DMEM (BersinBio) with 10% FBS (Biao Leibo) and cultured into the cell culture flasks. Next, the cell culture flasks were incubated in an incubator with 5% CO_2_ at 37℃. Cells passed between 4 and 10 generations were used for subsequent experiments. CAFs and PAFs were maintained in a complete medium (BersinBio).

To induce hypoxia, CAFs were cultured in the completed medium under a hypoxic atmosphere (94% N_2_, 5% CO_2_, and 1% O_2_) for 48 h (named H/CAFs). The control cells were cultured under normoxic environment for the same duration and were designated as N/CAFs.

### Cell culture

HCC cells (Huh7 and Hep3B) were procured from Procell (Wuhan, China). Cells were cultured in DMEM (BersinBio) supplemented with FBS (10%, Gibco).

### CD8^+^T separation

CD8^+^T cell isolation was performed using the CD8^+^T cell sorting kit (STEMERY, Fujian, China) according to the manufacturer’s instructions. Shortly, an appropriate amount of pre-cooled cell sorting solution was added to peripheral blood monocyte (PBMC), and then centrifuged (300 g) for 8 min. Then the supernatant was discarded, and the concentration of PBMC was adjusted to 10 × 10^8^ cells/mL by the proper amount of pre-cooled cell sorting solution. Then, 20 µL biotinylated antibody and 1 mL PBMC were fixed and incubated for 15 min (at 4℃). The supernatant in this experiment was discarded, and then streptavitin magnetic beads were added to PBMC according to the ratio of 20 µL magnetic beads and 1 mL PBMC. Finally, the CD8^+^T cells were obtained.

### Cell transfection

Short hairpin (sh) RNA (sh-NC) targeting SPI1 (sh-SPI1) and its negative control (sh-NC), as well as the v-Ha-ras Harvey rat sarcoma viral oncogene homolog (HRAS) overexpression plasmids (pcDNA-HRAS) and the corresponding empty vector (pcDNA), were synthesized by RiboBio (RiboBio Inc., Guangzhou, China). CAFs were infected with the lentivirus containing shRNAs using Lipomaster 3000 Transfection Reagent (Vazyme, Nanjing, China) according to the manufacturer’s instructions, and the cell culture medium (BersinBio) was collected. The Huh7 and Hep3B were transfected with plasmids using Lipomaster 3000 Transfection Reagent (Vazyme).

### Identification of PAFs and CAFs

PAFs and CAFs were isolated and identified. Firstly, cellular morphology was examined using a microscope (Olympus, Tokyo, Japan). Subsequently, fibroblast markers (Vimentin, α-smooth muscle actin (α-SMA), and fibroblast activation protein (FAP)), cytokeratin 19 recombinant protein (CK19), and E-cadherin expression levels were examined using western blot.

### Collection of cell culture medium

CAFs and PAFs were cultured in 25 cm^2^ cell culture flasks. The culture medium (BersinBio) was discarded when cells were cultured for 24 h. The cells were washed four times using phosphate buffer saline (PBS) (Biosharp, Beijing, China). Subsequently, the serum-free medium (BersinBio) was added to the cell culture flasks and incubated for 24 h. Next, the conditioned medium (CM) was collected and stored at −80℃. The CAFs-derived CM (CAFs-CM) and PAFs-derived CM (PAFs-CM) were used to treat Huh7 and Hep3B cells.

The 25 cm^2^ cell culture flasks were used to inoculate the CAFs, and the CAFs were transfected with sh-NC or sh-SPI1. Next, the culture medium (BersinBio) was discarded, and the medium (BersinBio) without serum was added to the cell culture flasks. After 24 h, the CM was obtained and named CAFs/sh-NC-CM and CAFs/sh-SPI1-CM. The Huh7 and Hep3B cells were treated with CAFs/sh-SPI1-CM and CAFs/sh-NC-CM.

### Western blot

Total proteins were separated using electrophoresis and transferred onto a polyvinylidene fluoride (PVDF) membrane (Vazyme). Subsequently, the PVDF membrane (Vazyme) was blocked at 37℃ for 2 h. After blocking, the PVDF membrane (Vazyme) was incubated with primary antibodies for 12 h at 4℃. The primary antibodies used were as follows: anti-E-cadherin (20874-1-AP, 1:5000, Chicago, IL, USA), anti-CK19 (10712-1-AP, 1:5000, Proteintech), anti-FAP (84018-4-AP, 1:4000, Proteintech), anti-α-SMA (14395-1-AP, 1:6000, Proteintech), anti-Vimentin (80232-1-AP, 1:6000, Proteintech), anti-β-actin (ab179467, 1:5000, Abcam, Cambridge, MA, USA), anti-programmed cell death protein-1 (PD-L1) (66248-1-Ig, 1:4000, Proteintech), anti-SPI1 (55100-1-AP, 1:1000, Proteintech), and anti-HRAS (18295-1-AP, 1:1000, Proteintech). Following primary antibody incubation, the membrane (PVDF, Vazyme) was incubated with secondary antibody (Proteintech) at 37℃ for 2 h. The protein bands were analyzed using the Tanon 1600 series multi-functional gel image analysis system (Tanon, Shanghai, China).

### Reverse-transcription quantitative polymerase chain reaction (qRT-PCR)

Total RNA was extracted using TRIzol solution (Solarbio, Beijing, China). After that, RNA concentration was assessed using the NanoDrop2000c (Thermo Fisher Scientific, Waltham, MA, USA). cDNA was synthesized using the PrimeScript™ RT reagent Kit (Takara, Osaka, Japan). The 7300 Real-Time PCR System (Applied Biosystems, Foster City, USA) was carried out for the qRT-PCR reaction. The sequences of forward and reverse primers used for qRT-PCR were as follows: SPI1, 5’-TGTTACAGGCGTGCAAAATGG-3’ and 5’-ACTGGACAGGAACAGGGTC-3’; HRAS, 5’-TCATTGATGGGGAGACGTGC-3’ and 5’-TCACCCGTTTGATCTGCTCC-3’; β-actin, 5’-CTTCGCGGGCGACGAT-3’ and 5’-ACTGGACAGGAACAGGGTC-3’.

### Glucose consumption analysis

Glucose consumption was measured using the glucose uptake assay kit (Abcam). The specific experimental procedures were performed according to the manufacturer’s instructions.

### Lactate production analysis

The lactate production was examined by the lactate assay (Dongjingdo, Shanghai, China). Briefly, HCC cells were seeded into 96-well microplates and subjected to the respective experimental treatments. Following treatment, the supernatants were collected and diluted 10 times with lactic acid buffer. 20 µL diluted sample and 80 µL lactic acid working solutions were added to each well of the 96-well microplate. After incubation at 37℃ for 30 min, the absorbance of each well was measured at 450 nm using the microplate reader (Thermo Fisher Scientific).

### Colony formation assay

Huh7 and Hep3B cells were cultured in 6-well microplates and subjected to the designated treatments. The cells were cultured in a humidified incubator with 5% CO_2_ at 37℃. After visible clones appeared in the petri dish, the culture medium (BersinBio) was discarded, and the cells were washed four times with PBS (Biosharp). Next, the cells were fixed with methanol (1 mL) for 16 min. After fixation, the cells were stained with crystal violet solution (0.1%, 1 mL/each well) (Biosharp) for 20 min. The proliferation was analyzed.

### Wound healing

Huh7 and Hep3B cells were treated with CAFs-CM and PAFs-CM and cultured in an incubator (Thermo Fisher Scientific) for 24 h. At approximately 85% confluence, a 20 µL pipette tip was used to generate a scratch in the monolayer. Next, the cells were washed four times with PBS (Biosharp) and then continued to be cultured in the incubator (Thermo Fisher Scientific). Finally, the migration of cells was analyzed using Image J software (version 1.54d; National Institutes of Health, Bethesda, Maryland, USA).

Huh7 and Hep3B cells were cultured into 6-well microplates. Then the cells (or cells transfected with plasmids) were co-cultured with CAFs/sh-SPI1-CM or CAFs/sh-NC-CM and the proliferation was analyzed. The rest of the experiment would follow the same steps as above.

### Transwell

The ability of invasion was determined using the pre-treated Matrigel (Beyotime) transwell chambers (Corning, Tewksbury, MA, USA). Following respective treatments, cells in serum-free medium (BersinBio) were added to the upper chamber, while the lower well was supplemented with medium containing 10% FBS (Gibco). Thereafter, the cells were fixed with paraformaldehyde (4%, Biosharp) for 30 min. After being fixed, the cells were stained with crystal violet (0.1%, Biosharp) for 20 min. Finally, the ability of invasion was examined using the microscope (Olympus).

### Flow cytometry

Huh7 and Hep3B cells were inoculated into 6-well microplates and then treated with different treatments. After washing the cells twice with PBS (Biosharp), pancreatic enzyme (1 mL, Biosharp) was added to digest and collect the cells. Next, the sample was mixed with PD-L1 antibody (ab205921, 1:1000, Abcam) in vortex and incubated for 15 min at 37℃ in the dark. PD-L1 expression levels were analyzed using the flow cytometry system (Beckman Coulter, Fullerton, CA, USA).

The apoptosis assay kit was purchased from Thermo Fisher Scientific. For the cell apoptosis assay, the Huh7 and Hep3B cells were cultured into 12-well microplates and subjected to the respective treatments. Subsequently, the other experimental procedures followed the instructions. Finally, the ability of cell apoptosis was examined using the flow cytometry system (Beckman Coulter).

### CD8^+^T proliferation assay

Huh7 and Hep3B cells were seeded into a 6-well microplate and subjected to the respective treatments, followed by co-culture with CD8^+^T. Subsequently, the cells were fixed with 4% paraformaldehyde (Biosharp) for 20 min (at 37℃). After fixation, the samples were incubated with anti-Ki-67 (14–5698-82, 1:100, Thermo Fisher Scientific) at 37℃ for 12 h, followed by incubation with HRP-labelled secondary antibody. After washing with PBS (Biosharp), the cell nucleus was stained with DAPI (Biosharp) for 5 min. The CD8^+^T proliferation was examined under the microscope (Olympus).

### Terminal deoxynucleotidyl transferase dUTP Nick-End labeling (TUNEL)

Apoptosis was examined using the TUNEL BrightGreen Apoptosis Detection Kit (Vazyme). Briefly, Huh7 and Hep3B cells were incubated in the 6-well microplate, subjected to the respective treatments, and co-cultured with CD8^+^T. After the cells were collected, paraformaldehyde (4%, Biosharp) was performed to fix the cells for 30 min. 100 µL of TUNEL detection solution (Vazyme) was added to each well (6-well plate) and incubated at 37℃ for 1 h in the dark. After the cells were washed four times, the DAPI (Biosharp) was used to treat the cell nucleus for 10 min. Ultimately, the apoptosis of CD8^+^T was detected under a microscope (Olympus).

### Bioinformatics

The CAFs-related dataset (GSE192912) was obtained from the Gene Expression Omnibus (GEO) database. *P* < 0.05 and logFC ≥ 1 were considered significant.

### Chromatin Immunoprecipitation (ChIP) assay

The CHIP assay was conducted using the ChIP Assay Kit (Beyotime) according to the manufacturer’s instructions. Briefly, the cells were collected. The sonication was used to fragment DNA, and then the cell lysate was incubated with anti-SPI1 (ab302623, 1:100, Abcam) at 4℃ overnight. IgG antibody (1:100, Abcam) was regarded as a negative control. The dynabeads protein G (Invitrogen) was used to incubate the samples for 2 h, and DNA enrichment was collected. The qRT-PCR reaction was performed for the CHIP assay.

### Dual-luciferase reporter assay

For the dual-luciferase reporter assay, mutant-type (mut) and wild-type (wt) reporter plasmids of HRAS sequences were amplified using PCR reaction and cloned into the PGL3 vector (GenePharma) and named mut-HRAS and wt-HRAS. Site-directed mutagenesis kit (Stratagene, CA, USA) was used to mutate the corresponding predicted binding site of SPI1 for HRAS. Afterwards, the 293 T were seeded into 12-well plates. When the cells reached a density of 70%, these cells were co-transfected with either mut-HRAS or wt-HRAS along with pcDNA or pcDNA-SPI1 for 24 h. Finally, the relative luciferase activities were examined using a dual-luciferase reporter assay system (Promega Corporation, Madison, WI, USA).

### Data analysis

Results were presented as the Mean ± standard deviation (SD). All statistical analyses were performed using GraphPad Prism 8 software (GraphPad Software, Boston, MA, USA). Comparisons between two groups were analyzed by Student’s *t*-tests, while differences among three or more groups were assessed by one-way analysis of variance (ANOVA). *P* ≤ 0.05 was considered statistically significant.

## Results

### CAFs-CM promotes proliferation, migration, invasion, glucose consumption, and lactate production in HCC cells

**Fig. 1:**
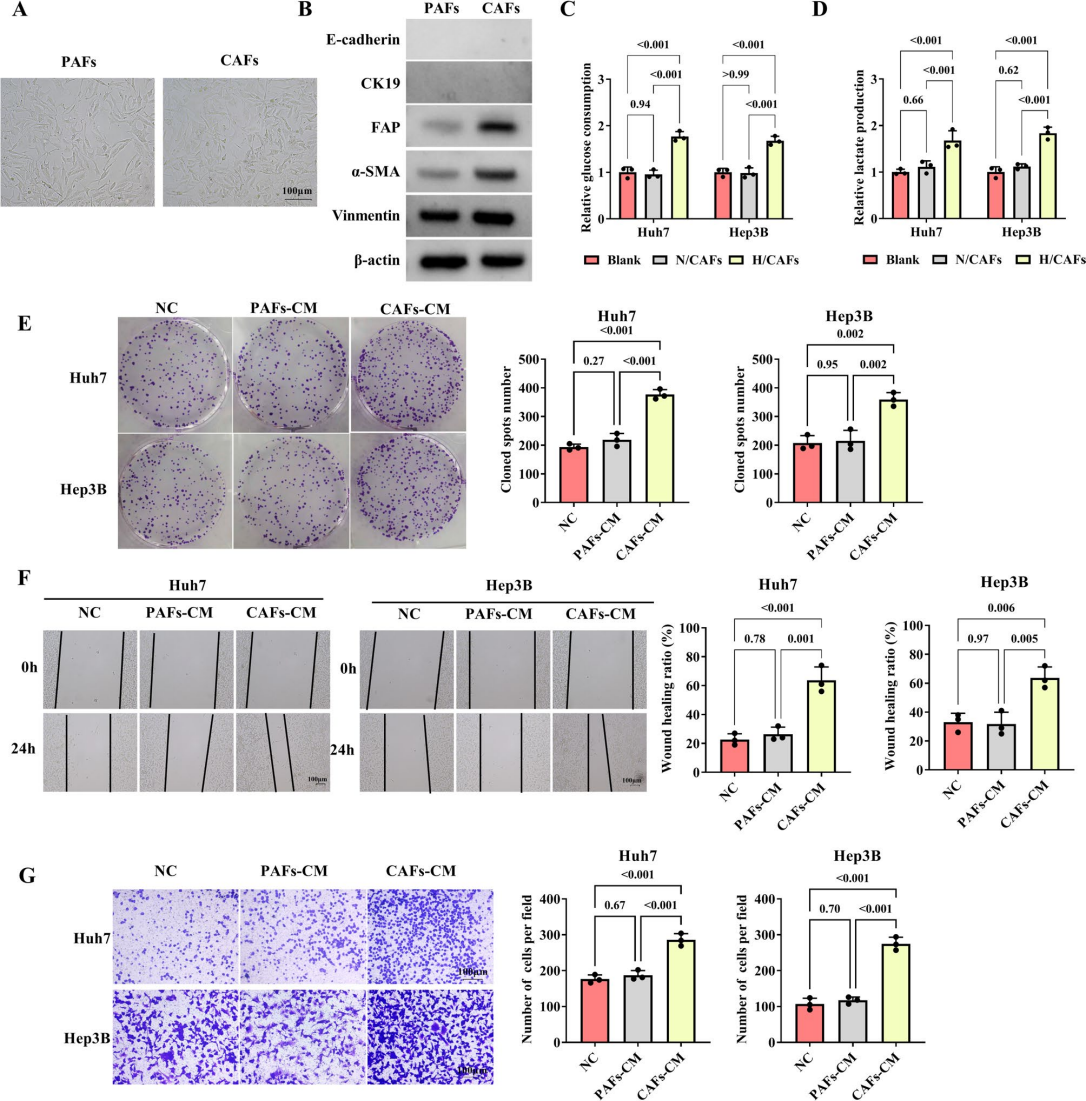
CAFs-CM expedites the proliferation, migration, invasion, glucose consumption, and lactate production in HCC cells. **A **Shapes of PAFs and CAFs. **B** Western blot was used to analyze the expression of α-SAM, FAP, Vimentin, E-cadherin, and CK19 in PAFs and CAFs. **C**-**D** In the Blank group (Huh7 or Hep3B), N/CAFs group (Huh7 or Hep3B cells were incubated with CAFs under normoxia), and H/CAFs group (Huh7 or Hep3B cells were incubated with CAFs under hypoxic stress), the glucose consumption and lactate production were examined by the glucose consumption and lactate production test kits, respectively. **E** In CAFs-CM-incubated HCC cells (Huh7 and Hep3B), PAFs-CM-incubated HCC cells, or normal-CM-incubated HCC cells, the cell proliferation was analyzed by the colony formation assay. **F**-**G** In CAFs-CM-incubated HCC cells (Huh7 and Hep3B), PAFs-CM-incubated HCC cells, or normal-CM-incubated HCC cells, the ability of migration and invasion was determined using wound healing and transwell assays, respectively

To investigate the role of CAFs in HCC tumorigenesis, CAFs and PAFs were isolated from HCC tissues and para-carcinoma tissues, respectively. Both CAFs and PAFs exhibited spindle-shaped morphology (Fig. 1A). The FAP, α-SMA, and the mesenchymal marker Vimentin are markers of fibroblasts. Western blot showed that α-SMA and Vimentin were expressed in CAFs and PAFs. Notably, the FAP, α-SMA, and Vimentin expression were up-regulated compared to PAFs, and the E-cadherin and CK19 were not expressed in CAFs and PAFs (Fig. 1B). Compared with N/CAFs, the glucose consumption was increased in H/CAFs (Fig. 1C). The lactate production level remained unchanged compared to the Blank group, whereas lactate production was enhanced in H/CAFs compared with N/CAFs (Fig. 1D). These findings underscore the crucial role of CAFs in the development of HCC. The cell proliferation was promoted in HCC cells incubated with CAFs-CM compared to HCC cells incubated with PAFs-CM (Fig. 1E). After being incubated with CAFs-CM for 24 h, the cell migration was facilitated compared to the HCC cells incubated with PAFs-CM (Fig. 1F). When compared to HCC cells incubated with PAFs-CM, the invasion ability was promoted in the HCC cells incubated with CAFs-CM (Fig. 1G). Based on these results, CAFs-CM contributes to the proliferation, migration, invasion, glucose consumption, and lactate production in HCC cells.

### CAFs-CM co-cultured with HCC cells inhibits HCC cell apoptosis and CD8^+^T proliferation and enhances CD8^+^T apoptosis

**Fig. 2:**
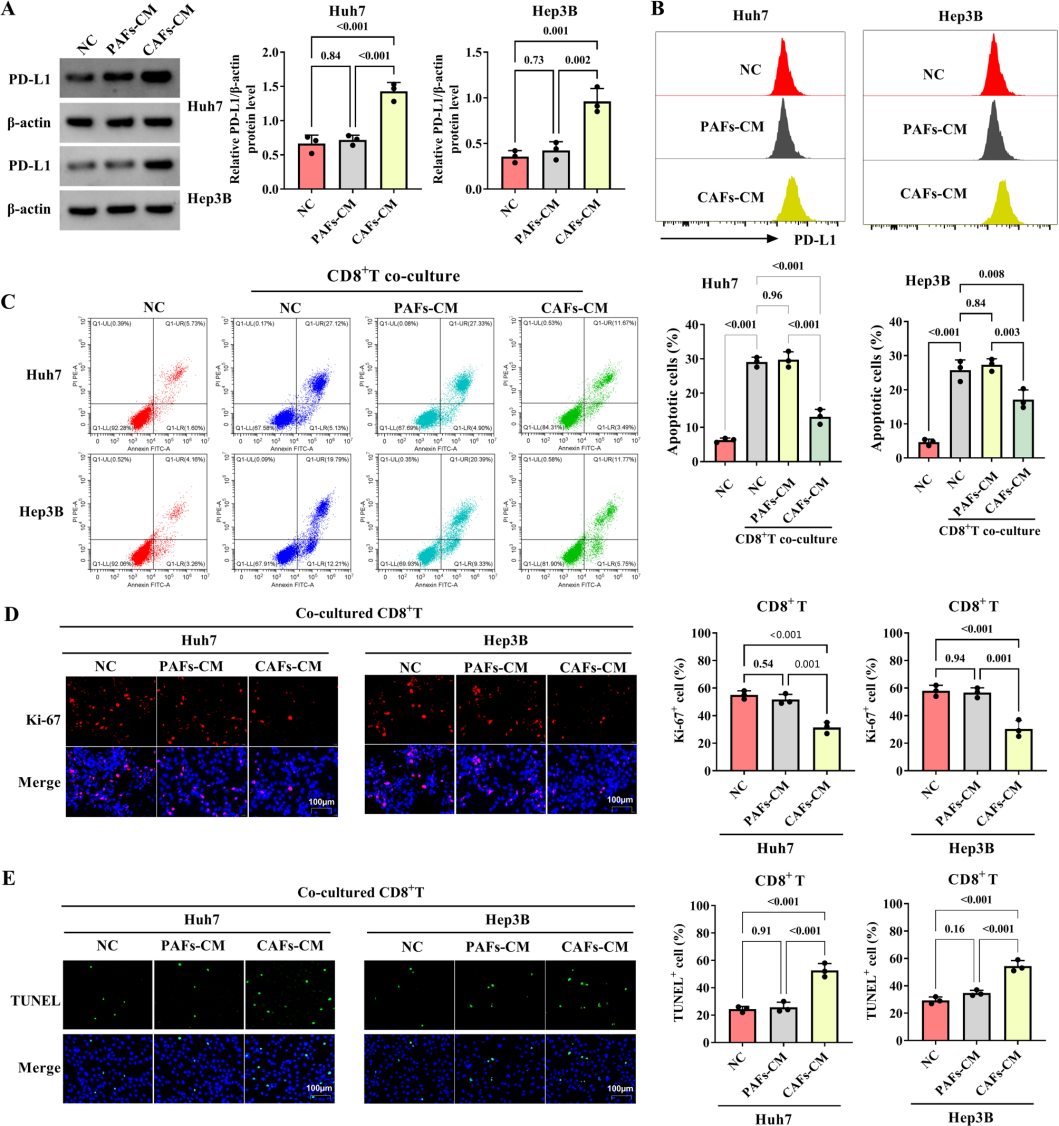
In HCC cells, PD-L1 levels and CD8^+^T apoptosis are promoted, and proliferation of CD8^+^T is inhibited by CAFs-CM. **A**-**B** In the NC group (HCC cells not treated), PAFs-CM group (HCC cells incubated with PAFs-CM), and CAFs-CM group (HCC cells incubated with CAFs-CM), the levels of PD-L1 were examined by western blot and flow cytometry, respectively. **C** The HCC cells were divided into four groups (NC group (HCC cells did not treat), NC (CD8^+^T co-culture) group, PAFs-CM (CD8^+^T co-culture) group, and CAFs-CM (CD8^+^T co-culture) group). The apoptosis of HCC cells was examined using flow cytometry. **D**-**E** The HCC cells were classified into three groups (CD8^+^T co-cultured with HCC cells (the HCC cells that were incubated with normal medium); CD8^+^T co-cultured with HCC cells (the HCC cells that were incubated with CAFs-CM); and CD8^+^T co-cultured with HCC cells (the HCC cells that were incubated with PAFs-CM).The proliferation and apoptosis of CD8^+^T were detected by immunofluorescence and TUNEL assays, respectively

The role of CAFs-CM in immune escape of HCC cells was analyzed. PD-L1 plays a crucial role in maintaining self-tolerance and immune homeostasis [19]. As shown in Fig. 2A, the western blot revealed that the expression of PD-L1 was increased in HCC cells treated with CAFs-CM. Flow cytometry assay showed the highly expressed PD-L1 in HCC cells treated with CAFs-CM compared to PAFs-CM group (Fig. 2B). When CD8^+^T were co-cultured with HCC cells treated with CAFs-CM, the apoptosis of HCC cells was inhibited compared with CD8^+^T co-cultured with HCC cells treated with PAFs-CM (Fig. 2C). In CD8^+^T co-cultured with HCC cells, the Ki-67 + cells were decreased by CAFs-CM (Fig. 2D). The apoptosis of CD8^+^T was promoted in HCC cells treated with CAFs-CM when compared to HCC cells treated with PAFs-CM (Fig. 2E). Above, in HCC cells, CAFs-CM promotes PD-L1 expression levels and promotes apoptosis of CD8^+^T.

###  The differentially expressed gene (DEG) SPI1 is identified in PAFs and CAFs

**Fig. 3:**
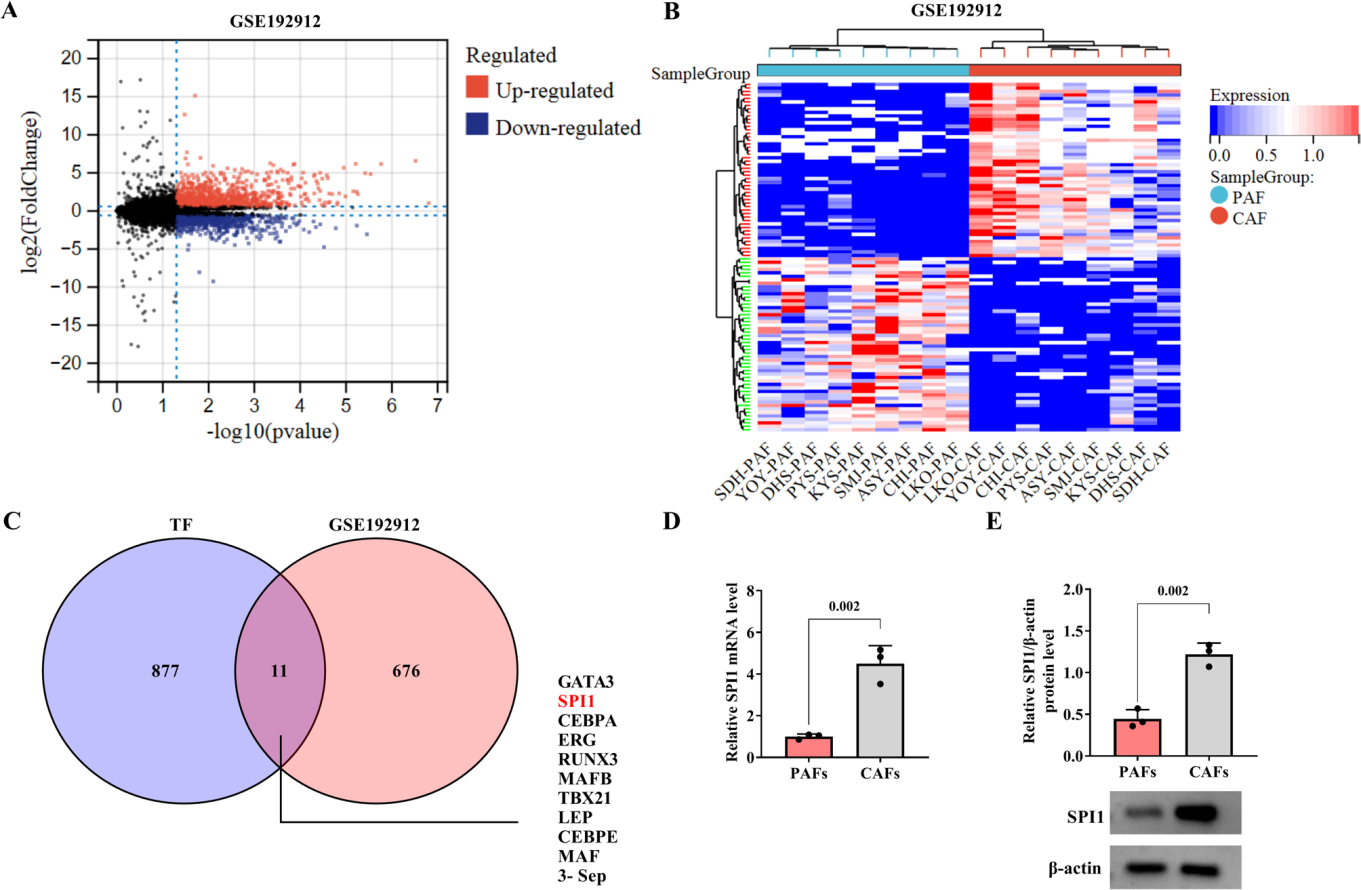
The DEGs in PAFs and CAFs are analyzed. **A** The volcano map was performed to analyze the DEGs in PAFs and CAFs based on the GEO database. *P* < 0.05 and logFC ≥ 1 were considered significant. **B** In the GSE192912 dataset, the genes of up-regulation and down-regulation in top 50 DEGs were analyzed by heat map. **C** The common DEGs in GSE192912 and transcription factors were screened via GEO and hTFtarget databases. **D** The SPI1 expression in CAFs and PAFs was examined by qRT-PCR. **E** Western blot was performed to examine the expression of SPI1 in CAFs and PAFs

A volcano map was generated to analyze the DEGs between PAFs and CAFs. A total of 1221 DEGs (including 687 up-regulated genes and 534 down-regulated genes) were identified in PAFs and CAFs (Fig. 3A). The heat map displayed the top 50 up-regulated or down-regulated genes in the GSE192912 dataset (Fig. 3B). As shown in Fig. 3C, the Venn diagram exhibited there were 11 common genes from the intersection of the up-regulated DEGs in the GSE192912 dataset and a set of transcription factors (such as SPI1, GATA3, and so on). SPI1 regulated liver metabolic function by affecting macrophage polarization [20]. Therefore, authors propose SPI1 may play an important role in HCC cells and immune escape. Subsequently, the qRT-PCR and western blot results demonstrated that SPI1 expression was increased in CAFs (Fig. 3D, E). Hence, SPI1 expression is up-regulated in CAFs.

### In CAFs-CM-cultured HCC cells, SPI1 contributes to glucose consumption, lactate production, proliferation, invasion, and immune escape

**Fig. 4:**
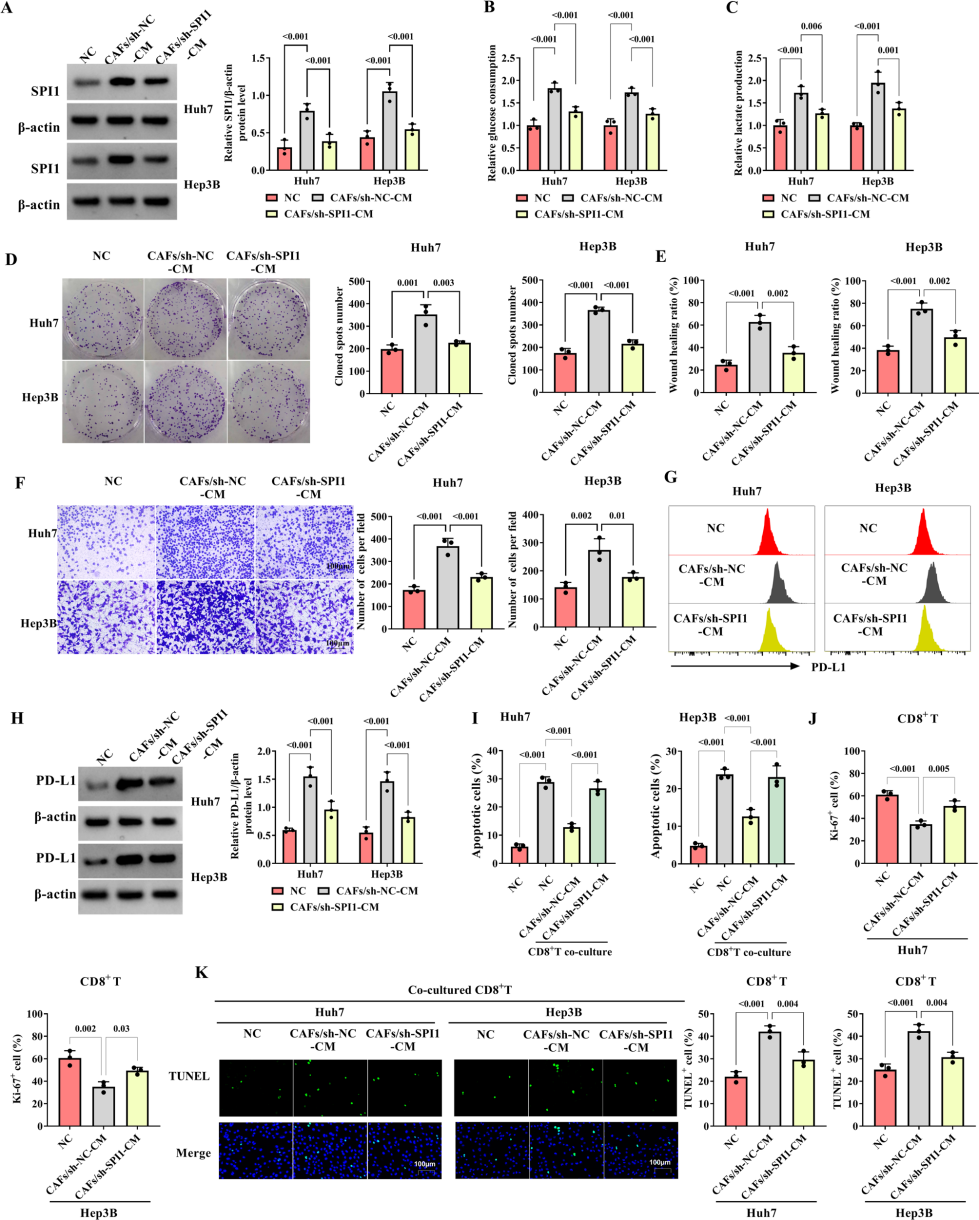
In CAFs-CM-cultured with HCC cells, SPI1 knockdown inhibits PD-L1 levels, glucose consumption, lactate production, proliferation, invasion, and immune escape. The HCC cells were divided into six groups according to the different treatments (NC group (HCC cells), CAFs/sh-NC-CM group (HCC cells were incubated with CAFs transfected with sh-NC), CAFs/sh-SPI1-CM group (HCC cells were incubated with CAFs transfected with sh-SPI1), NC co-cultured CD8^+^T group (HCC cells co-cultured with CD8^+^T), CAFs/sh-NC-CM co-cultured CD8^+^T group (after HCC cells were incubated with CAFs transfected with sh-NC, co-cultured with CD8^+^T), and CAFs/sh-SPI1-CM co-cultured CD8^+^T group (after HCC cells were incubated with CAFs transfected with sh-SPI1, co-cultured with CD8^+^T)). **A** The expression of SPI1 was determined by western blot. **B**-**C** The glucose consumption and lactate production detection kits were performed to analyze the glucose consumption and lactate production. **D** The proliferation was examined using colony assay. **E** The migration was detected using wound healing. **F** The transwell assay was used to analyze the ability of invasion. **G**-**H** The expression of PD-L1 was detected by flow cytometry and western blot, respectively. **I** The apoptosis of HCC cells was examined by flow cytometry. **J** The CD8^+^T proliferation was analyzed using immunofluorescence. **K** The TUNEL assay was performed to analyze the apoptosis of CD8^+^T

To explore the role of SPI1 in CAFs-CM-cultured HCC cells, the cell functions were examined. Firstly, the CAFs-CM increased SPI1 expression in HCC cells, which was abrogated by sh-SPI1 (Fig. 4A). In HCC cells, glucose consumption and lactate production were promoted by CAFs-CM, but silencing SPI1 weakened these actions (Fig. 4B, C). Compared with the NC group, the proliferation was facilitated in HCC cells incubated with CAFs-CM, nevertheless, SPI1 knockdown undermined the effect (Fig. 4D). The cell migration and invasion were promoted in HCC cells incubated with CAFs-CM, however, the action was weakened by sh-SPI1 (Fig. 4E, F). Flow cytometry and western blot results demonstrated that CAFs-CM up-regulated the expression of PD-L1 in HCC cells, while down-regulated SPI1 alleviated this effect (Fig. 4G, H). In HCC cells incubated with normal-CM co-cultured with CD8^+^T, the apoptosis of HCC cells was reinforced, and CAFs inhibited HCC cell apoptosis, while this action was ameliorated by SPI1 knockdown (Fig. 4I). The CD8^+^T proliferation was suppressed in HCC cells incubated with CAFs-CM, but sh-SPI1 weakened the effect (Fig. 4J). TUNEL assay showed that CAFs-CM increased the CD8^+^T apoptosis rate in HCC cells co-cultured with CD8^+^T group, which was abrogated by sh-SPI1 (Fig. 4K). By thinking about the above results, in CAFs-CM-cultured HCC cells, sh-SPI1 inhibits PD-L1 levels, glucose consumption, lactate production, proliferation, invasion, and immune escape.

### SPI1 enhances HRAS levels via promoting HRAS transcription

**Fig. 5:**
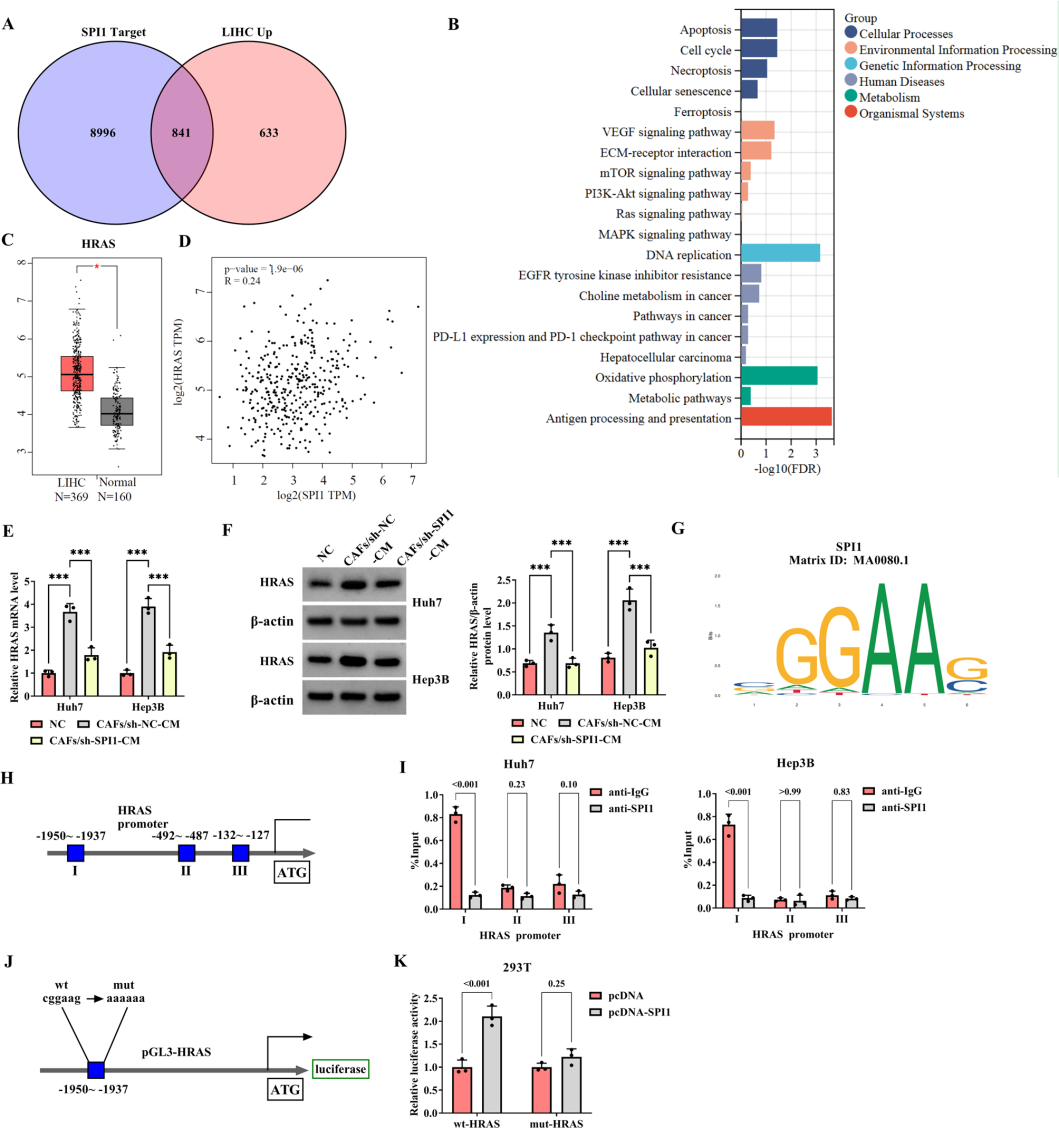
SPI1 facilitates HRAS levels by promoting HRAS transcription. **A** The Venn diagram of DEGs. A comparison between 9837 SPI1 targets and 1474 LIHC up-regulated genes (LIHC Up) revealed 841 common targets. **B** KEGG analysis of the common targets. **C** HRAS expression in Normal and LIHC tissues from the GEPIA database. **D** Correlation analysis of HRAS and SPI1 expression in LIHC from the GEPIA database. **E**-**F** The HCC cells were divided into three groups and named NC (HCC cell), CAFs/sh-NC-CM (HCC cells cultured with the CM collected from the CAFs transfected with sh-NC), and CAFs/sh-SPI1-CM (HCC cells cultured with the CM collected from the CAFs transfected with sh-SPI1). The HRAS mRNA and protein levels were examined by qRT-PCR and western blot, respectively. **G** The transcription factor SPI1 binding site was predicted by JASPAR. **H** HRAS promoter regions were predicted by JASPAR. (I) The CHIP assay was used to detect the binding between SPI1 and HRAS promoters. **J** The schematic diagram showed the binding sites of SPI1 for the promoter region of the HRAS gene. **K** The association between SPI1 and HRAS was analyzed using dual-luciferase reporter assay

Next, this study investigated the mechanism by which SPI1 acts in HCC cells incubated with CAFs-CM. The Venn diagram showed that there were 841 common targets in the intersection of the SPI1 target genes and the up-regulated genes in LIHC (Fig. 5A). Subsequently, the Kyoto Encyclopedia of Genes and Genomes (KEGG) enrichment analysis was performed on these 841 genes, and the results demonstrated that the target genes were enriched in the Apoptosis, PD-L1 expression and PD-1 checkpoint pathway in cancer, mitogen-activated protein kinase (MAPK) signaling pathway, and Ras signaling pathway (Fig. 5B). In the GEPIA database, HRAS expression was up-regulated in LIHC (Fig. 5C) and HRAS expression was positively correlated with SPI1 expression (Fig. 5D). CAFs increased the HRAS levels in HCC cells, which were weakened by SPI1 knockdown (Fig. 5E, F). As shown in Fig. 5G and H, the transcription factor SPI1 could bind to HRAS promoters, which was predicted using the JASPAR database. CHIP and the dual-luciferase reporter assays showed that SPI1 could bind to HRAS (Fig. 5I-K). Based on these results, SPI1 enhances HRAS expression by promoting its transcription.

### SPI1 derived from CAFs expedites proliferation, migration, invasion, glucose consumption, lactate production, and immune escape by elevating HRAS levels in HCC cells

**Fig. 6:**
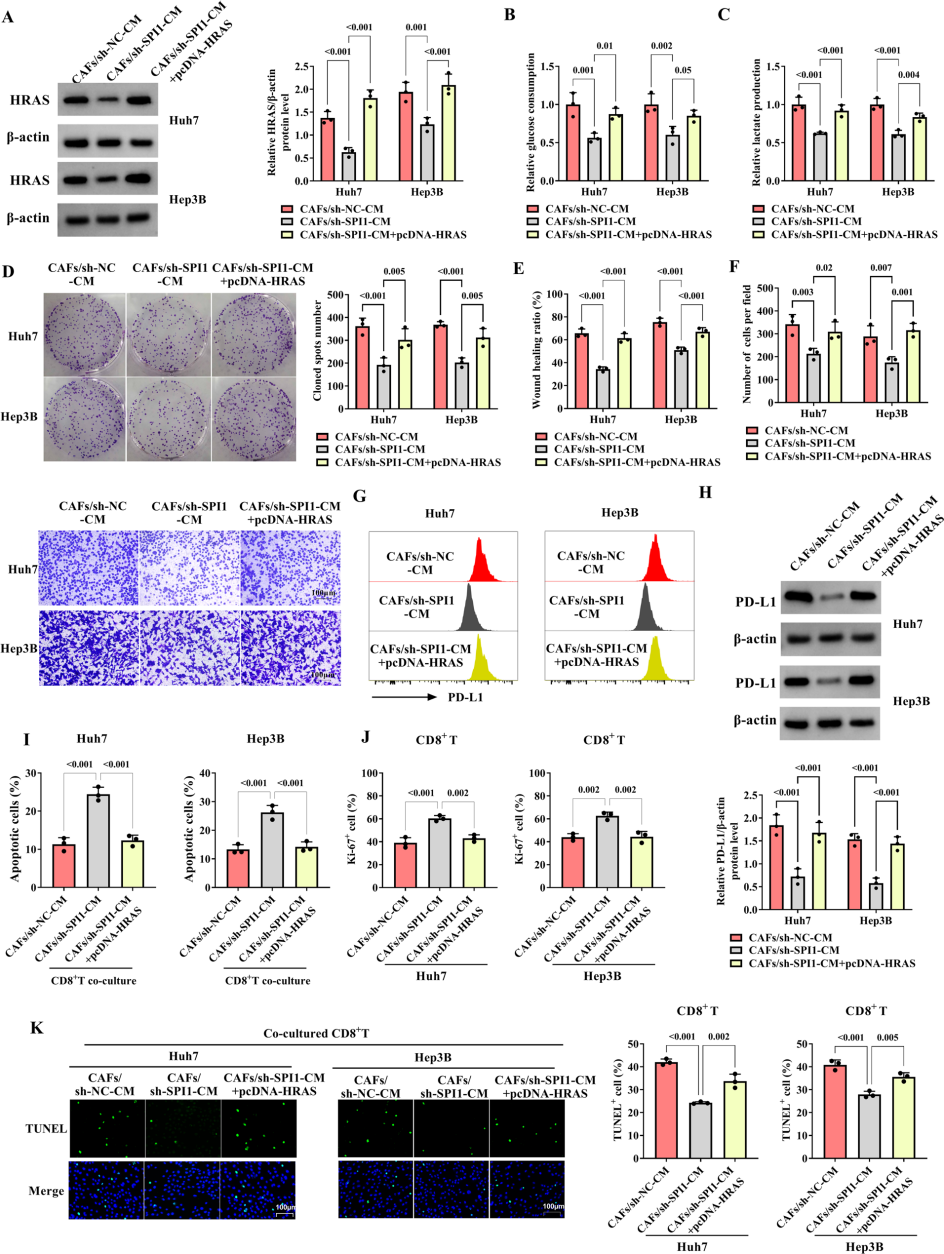
SPI1 derived from CAFs inhibits the progression of HCC by inhibiting HRAS levels. The HCC cells were divided into three groups and named CAFs/sh-NC-CM (CM of CAFs transfected with sh-NC was used to culture with HCC cells), CAFs/sh-SPI1-CM (after CAFs transfected with sh-SPI1, the CM was used to culture with HCC cells), and CAFs/sh-NC-CM + pcDNA-HRAS (after CAFs transfected with sh-SPI1, the CM was used to culture with HCC cells transfected with pcDNA-HRAS). **A** The HRAS expression was analyzed by western blot. **B**-**C** The glucose consumption and lactate production were examined by glucose consumption and lactate production test kits. **D** The colony assay was performed to detect the cell proliferation. **E** The ability of migration was examined by wound healing. **F** The cell invasion was determined using transwell assays. **G**-**H** PD-L1 expression was measured by flow cytometry and western blot, respectively. **I** The cell apoptosis of HCC cells was estimated by flow cytometry. **J** The proliferation ability of CD8^+^T was analyzed by immunofluorescence. **K** The TUNEL assay was utilized to examine the cell apoptosis of CD8^+^T

Finally, researchers demonstrated the role of SPI1 and HRAS in HCC cells cultured with CAFs-CM. As shown in Fig. 6A, compared with the CAFs/sh-NC-CM group, HRAS levels were decreased in HCC cells cultured with CAFs-CM transfected with sh-SPI1, while up-regulated HRAS weakened this action. In HCC cells cultured with CAFs-CM transfected with sh-SPI1, the glucose consumption and lactate production were inhibited compared with the CAFs/sh-NC-CM group, which was weakened by HRAS overexpression (Fig. 6B, C). The ability of proliferation in HCC cells cultured with CAFs-CM was decreased by SPI1 knockdown, while up-regulated HRAS overturned this effect (Fig. 6D). Silencing SPI1 suppressed cell migration and invasion of HCC cells cultured with CAFs-CM, but HRAS overexpression abolished this action (Fig. 6E, F). After HCC cells were cultured with CAFs-CM transfected with sh-SPI1, the expression of PD-L1 was inhibited compared with CAFs/sh-NC-CM, HRAS up-regulation abolished this effect (Fig. 6G, H). HCC cell apoptosis was promoted in CAFs/sh-SPI1-CM (CD8^+^T co-culture) group, which was abrogated by HRAS overexpression (Fig. 6I). In HCC cells with CAFs-CM transfected to sh-NC co-cultured with CD8^+^T, the proliferation of CD8^+^T was promoted and CD8^+^T apoptosis was suppressed by down-regulated SPI1, while HRAS overexpression weakened and overturned these effects (Fig. 6J, K). To sum up, SPI1 derived from CAFs induces the malignant activities of HCC cells by regulating HRAS levels.

## Discussion

HCC pathogenesis is a complex, multi-factorial process fundamentally driven by an imbalance between the inactivation and activation of tumor suppressor genes and the activation of oncogenes [21]. The global incidence of HCC demonstrates to sustained increase, resulting in substantial economic losses amounting to billions of dollars annually imposing a severe burden on families and societies worldwide [22, 23]. A deeper understanding of the underlying molecular pathology of HCC is therefore imperative.

CAFs serve as key constituents in diverse tumor microenvironments, including that of HCC. The TME in HCC is notably abundant in CAFs, which secrete an excess of chemokines, cytokines, and growth factors, thereby fostering tumor cell proliferation and invasion [24]. Within HCC, CAFs predominantly originate from hepatic stellate cells. These cells can drive HCC progression through various mechanisms, such as promoting tumor cell proliferation, enhancing the stemness of HCC cells, stimulating angiogenesis, inducing ECM remodeling, and facilitating the formation of an immunosuppressive TME [25–27]. In this study, compared to N/CAFs, the glucose consumption and lactate production were higher in H/CAFs. Moreover, CAFs-CM promoted cell proliferation, migration, and invasion in HCC. Furthermore, Shalapour et al. identified a population of IgA^+^ plasma cells, a type of regulatory B cell that accumulated in the liver and suppressed CD8^+^T cell response via PD-L1 expression [28]. Additionally, endothelin-positive CAFs in HCC have been shown to recruit and induce macrophage M2 polarization, thereby advancing HCC progression [29]. Interestingly, our results revealed that the CAFs-CM accelerated the apoptosis of CD8^+^T and increased PD-L1 levels and inhibited proliferation of CD8^+^T. These findings suggested that CAFs-CM not only contribute to HCC progression and immune escape but also that hypoxic-induced CAFs facilitate glycolysis.

To elucidate the underlying mechanism of action of CAFs-CM, this study conducted further bioinformatics analysis, and the results exhibited that SPI1 levels were increased in CAFs from the GSE192912 dataset. Based on this finding, researchers hypothesized that CAFs might promote HCC progression through SPI1. SPI1, a key transcriptional regulator [30], can suppress myeloma growth through its capacity to inhibit the transcription of interferon regulatory factor 4 [31]. A study demonstrated that recombinant PR domain containing protein 1/B lymphocyte-induced maturation protein 1 orchestrates cancer immune evasion in HCC cells by regulating the ubiquitin specific peptidase 22-SPI1-PD-L1 axis [32]. Our data showed that silencing SPI1 could reverse CAFs-CM-induced the malignant progression of HCC and immune escape. Additionally, HRAS interacted with SPI1. HRAS enhanced proliferation and invasion of HCC by upregulating heat shock protein family B (small) member 1 [33]. Moreover, up-regulated wild-type HRAS drove non-alcoholic steatohepatitis to HCC in mice [34]. SPI1 derived from CAFs promoted HCC progression by up-regulating HRAS expression.

Collectively, our results demonstrate that CAF-derived SPI1 promotes progression of HCC and immune escape via interacting with HRAS. These findings may identify new biomarkers or targets for early diagnosis and HCC treatment, suggesting new therapeutic approaches for HCC.

## Supplementary Information

Supplementary Material 1.

Supplementary Material 2.

## Data Availability

The datasets used and analysed during the current study are available from the corresponding author on reasonable request.
