# Supplementary material for: CAFs-derived SPI1 in tumor fibroblasts promotes malignant behaviors of liver cancer cells and immune escape by regulating HRAS and PD-L1 transcription
Source: Hereditas. 2025 Nov 26;162:233. doi: 10.1186/s41065-025-00605-2 (PMC12659378; doi:10.1186/s41065-025-00605-2)

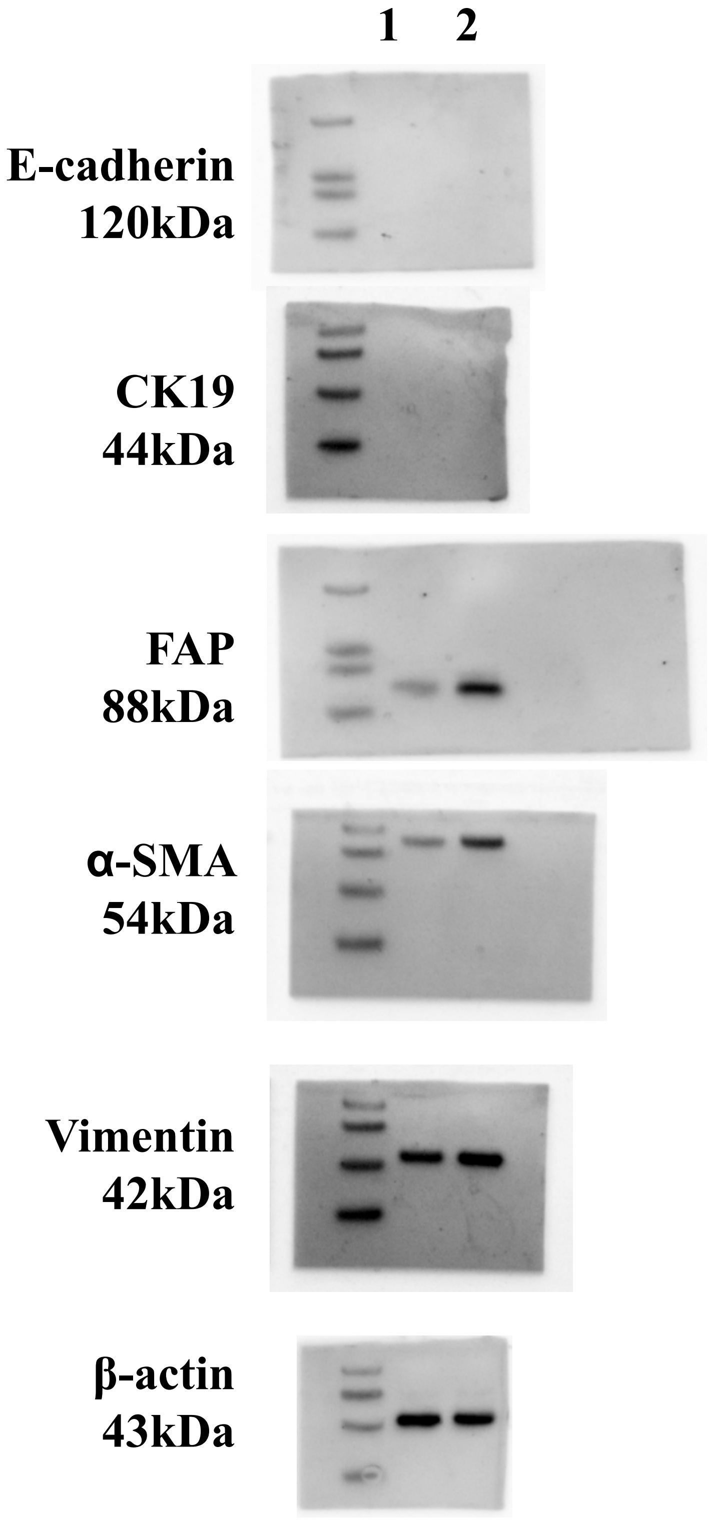

Fig (1B)

1 PAFs

2 CAFs

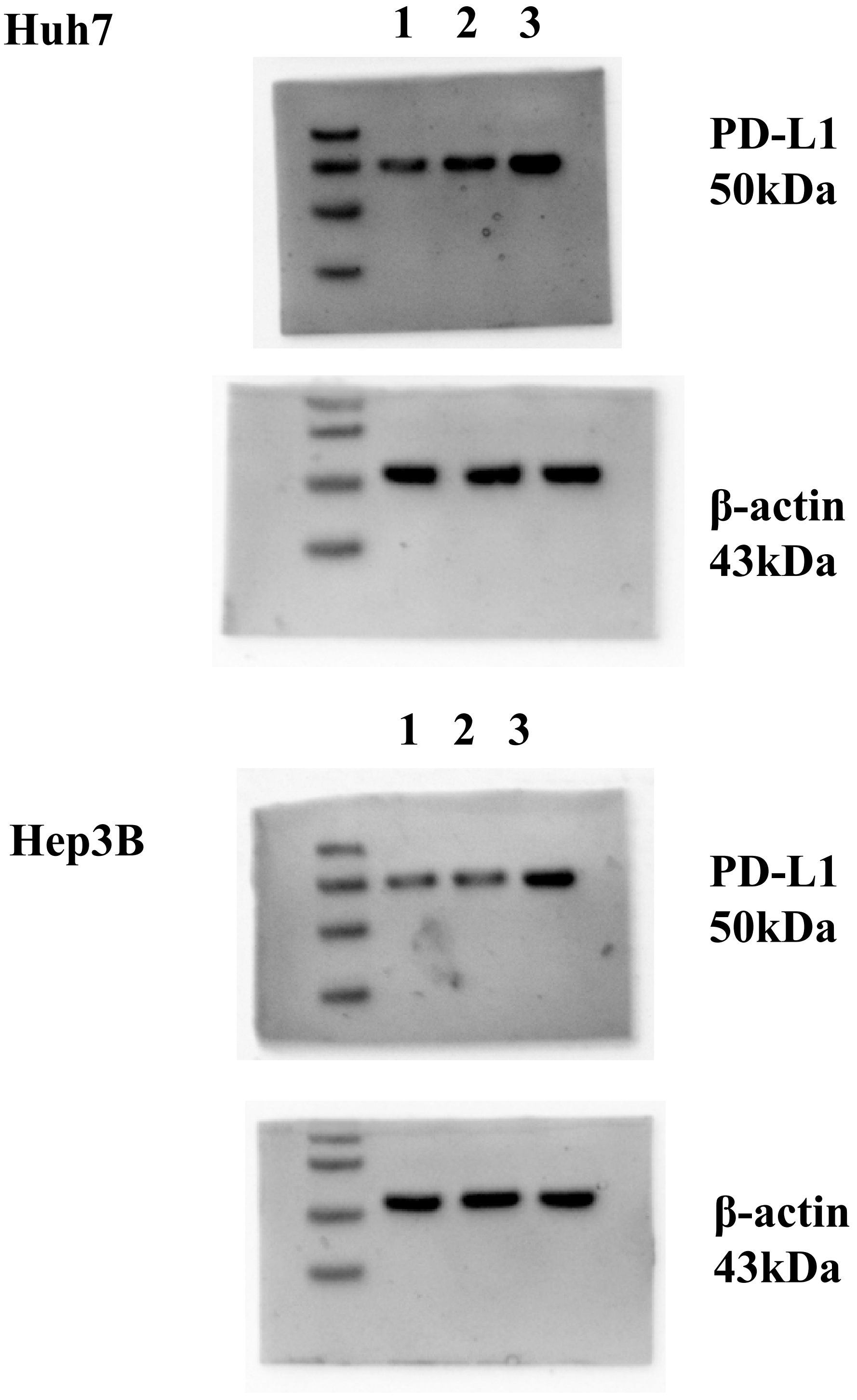

Fig (2A)

1 NC

2 PAFs-CM

3 CAFs-CM

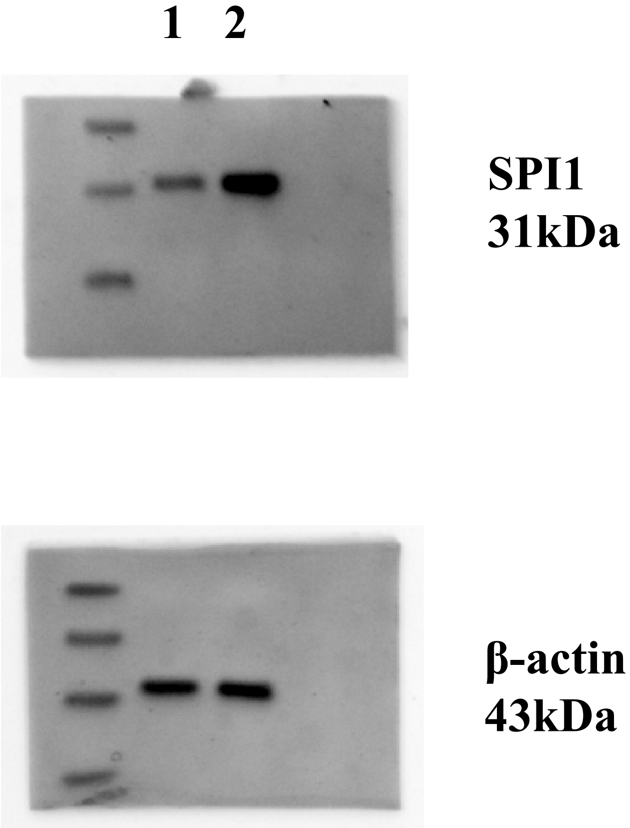

Fig (3E) 1 PAFs

2 CAFs

Huh7

1 2 3

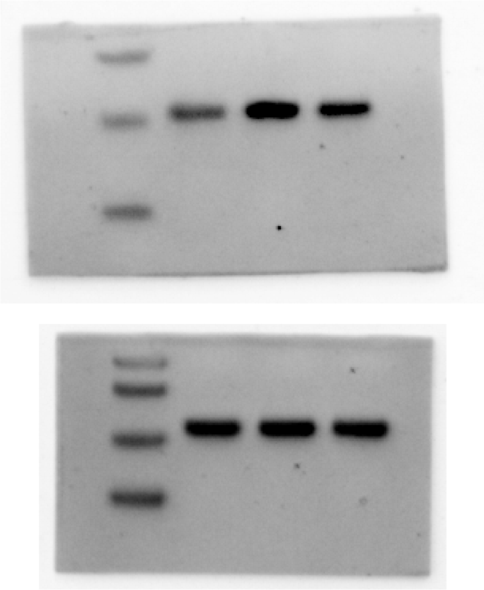

SPI1
31kDa

β-actin
43kDa

1 2 3

Hep3B

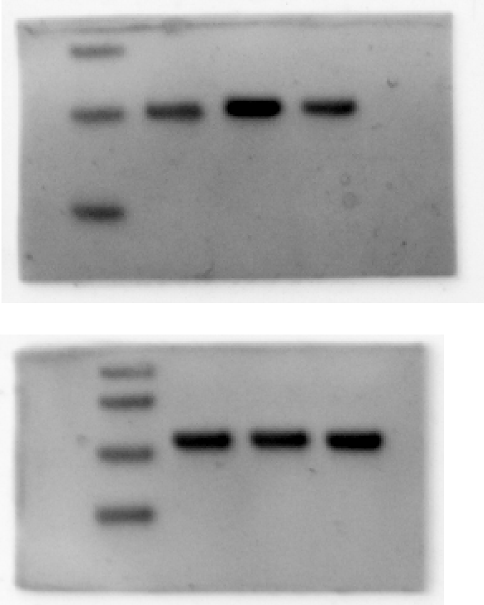

SPI1
31kDa

β-actin
43kDa

Fig (4A)

1 NC

2 CAFs/sh-NC-CM

3 CAFs/sh-SPI1-CM

Huh7

1 2 3

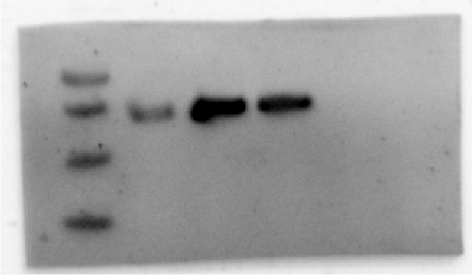

PD-L1
50kDa

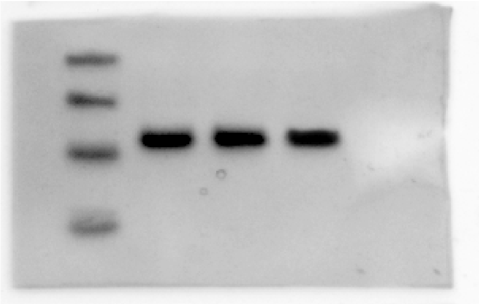

β-actin
43kDa

Hep3B

1 2 3

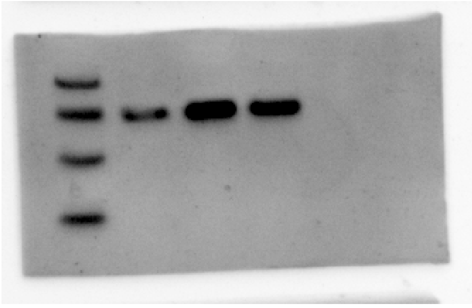

PD-L1
50kDa

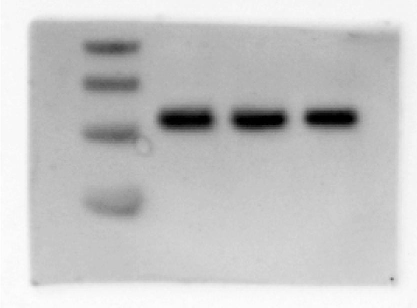

β-actin
43kDa

Fig (4H)

1 NC

2 CAFs/sh-NC-CM

3 CAFs/sh-SPI1-CM

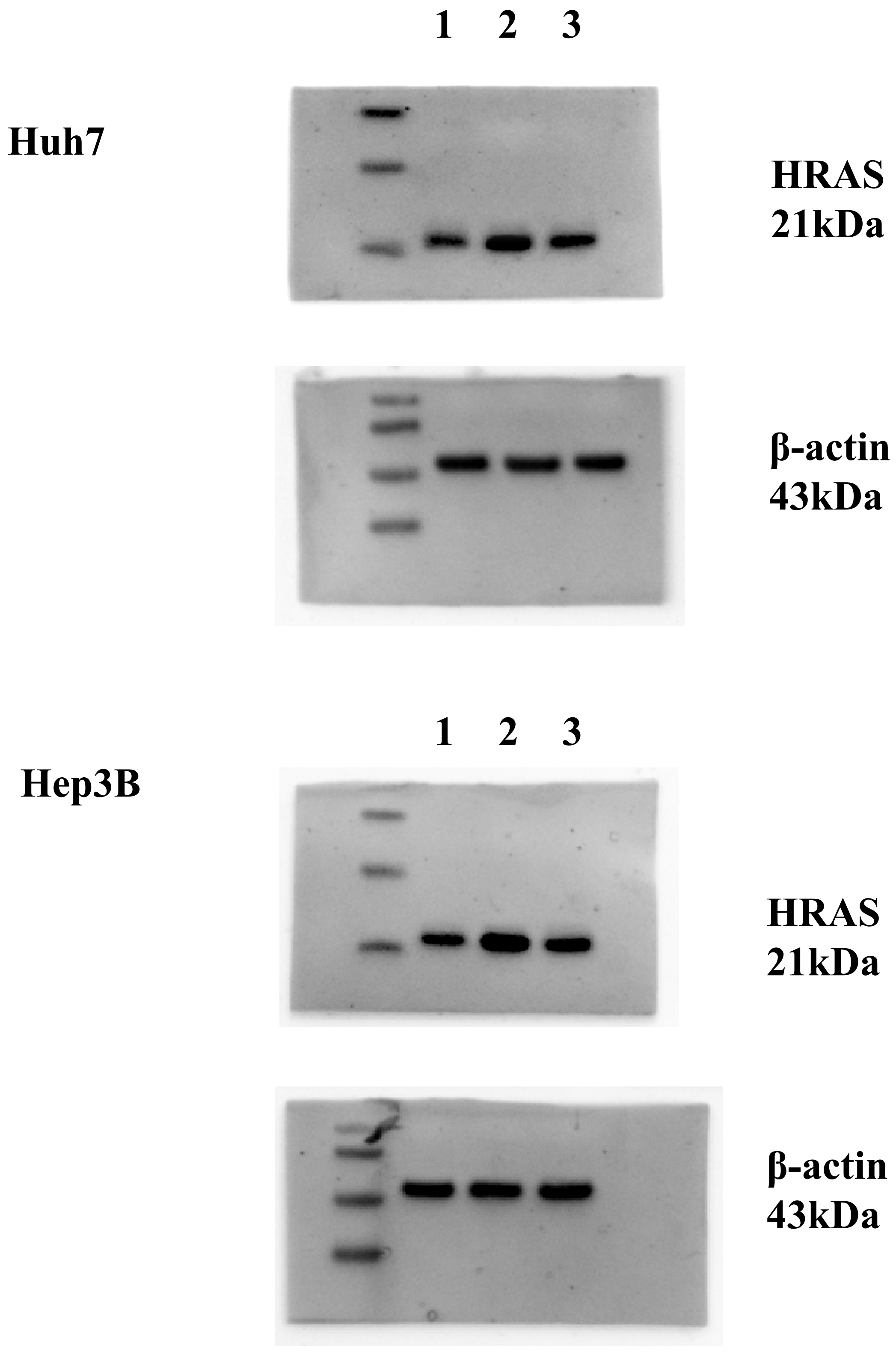

Fig (5F)

1 NC

2 CAFs/sh-NC-CM

3 CAFs/sh-SPI1-CM

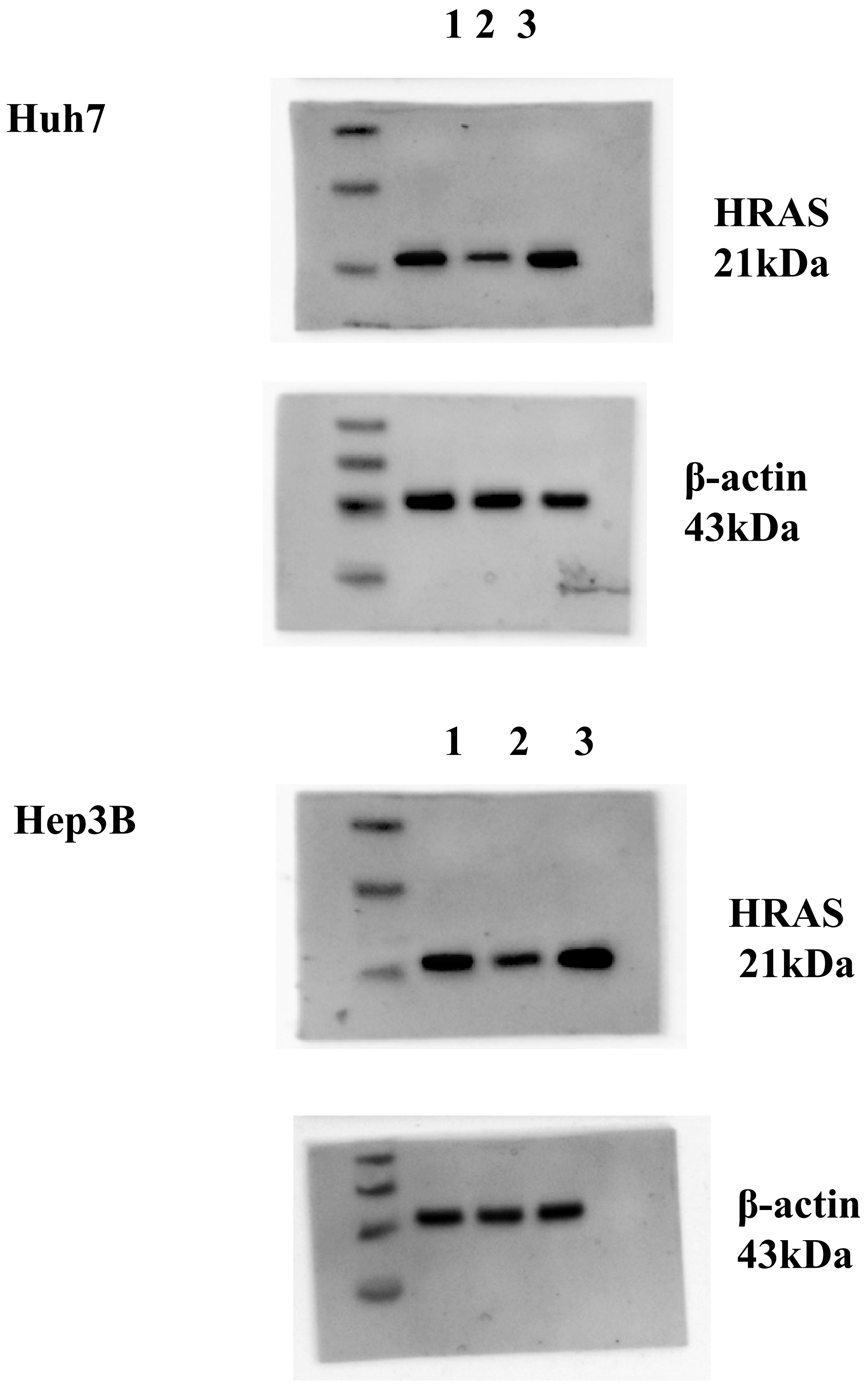

Fig (6A) 1 CAFs/sh-NC-CM

2 CAFs/sh-SPI1-CM

3 CAFs/sh-SPI1-CM+pcDNA-HRAS

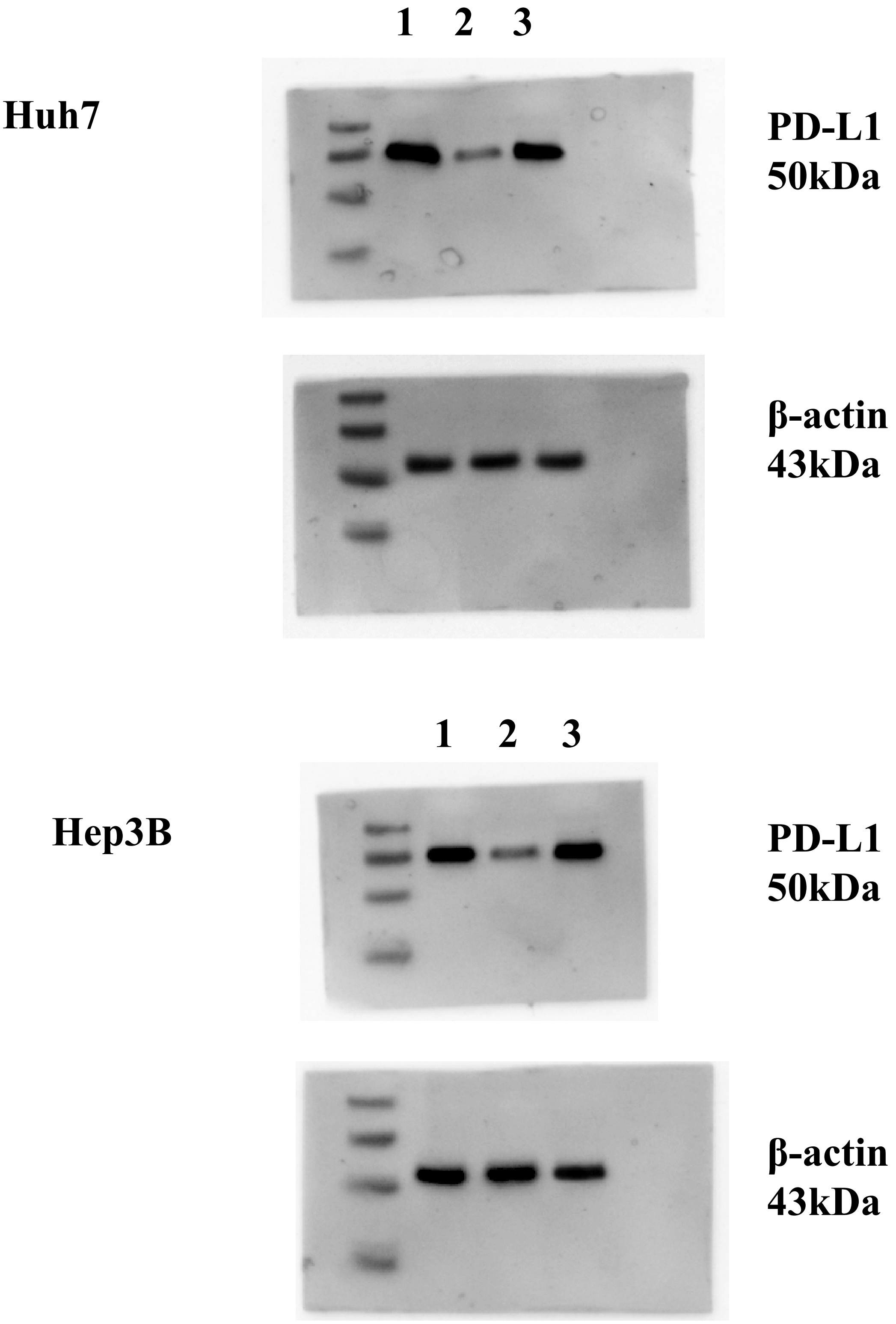

Fig (6H)

1 CAFs/sh-NC-CM

2 CAFs/sh-SPI1-CM

3 CAFs/sh-SPI1-CM+pcDNA-HRAS

- **The samples derive from the same experiment and that gels/blots were processed in parallel.**

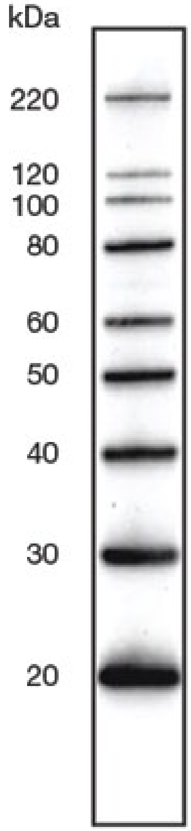

Supplement: Supplementary file 1 — Supplementary Material 1. [file 41065_2025_605_MOESM1_ESM.pdf]
