# Supplementary figures and images for: CAFs-derived SPI1 in tumor fibroblasts promotes malignant behaviors of liver cancer cells and immune escape by regulating HRAS and PD-L1 transcription
Source: Hereditas. 2025 Nov 26;162:233. doi: 10.1186/s41065-025-00605-2 (PMC12659378; doi:10.1186/s41065-025-00605-2)

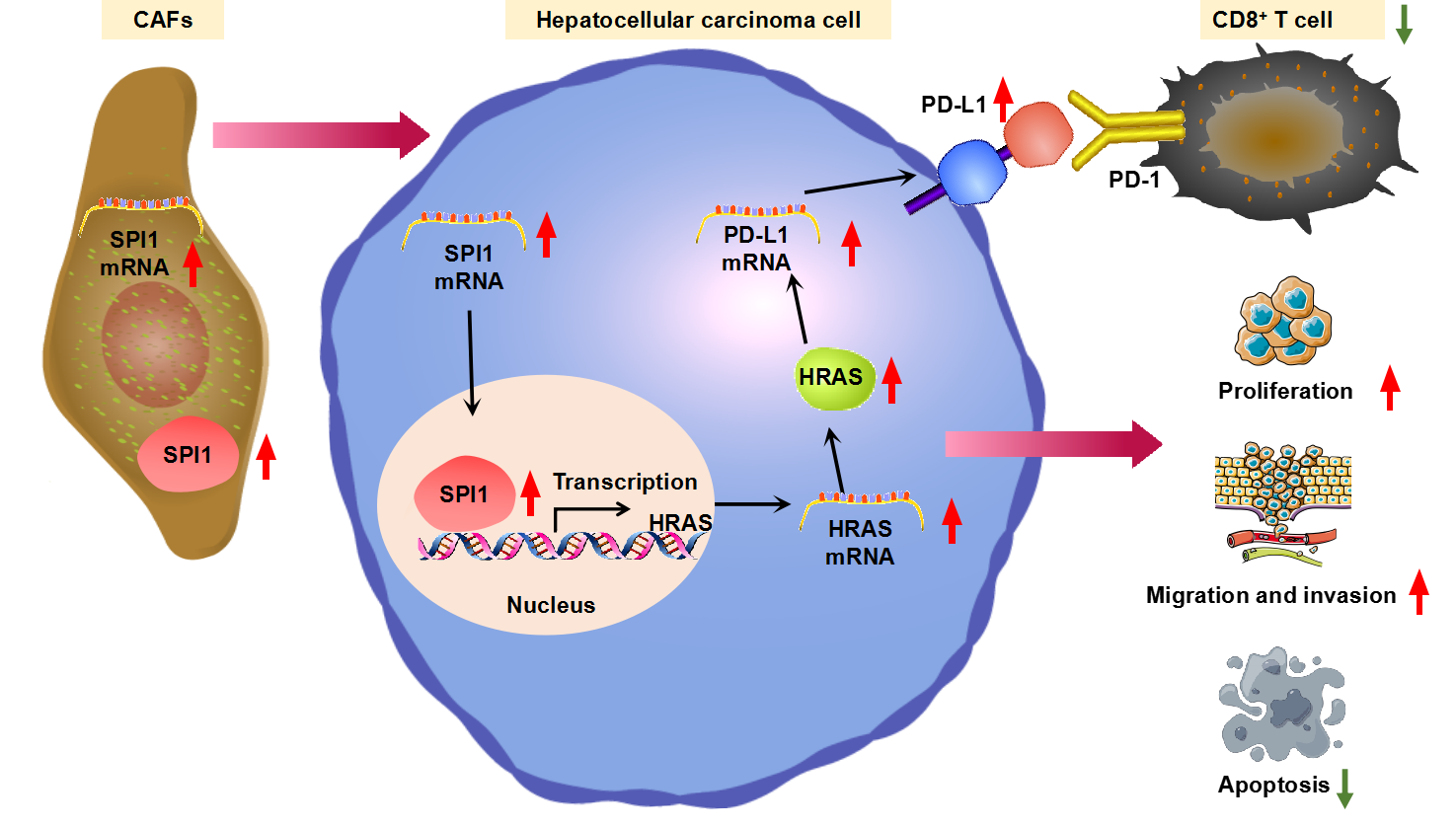

Supplement: Supplementary file 2 — Supplementary Material 2. [file 41065_2025_605_MOESM2_ESM.tif]
